# How Shall We Treat Hemorrhoids?

**Published:** 1888-03

**Authors:** Jno. Thad. Johnson

**Affiliations:** Atlanta, Ga.


THE ATLANTA MEDICAL AND SURGICAL JOURNAL.

VOL. V. MARCH, 1888. No. 1.

### Original Communications.

### HOW SHALL WE TREAT HEMORRHOIDS?

BY JNO. THAD. JOHNSON, M. D., ATLANTA, GA.

In selecting a method of operation when taking charge of a case of hemorrhoids, there are several plans presented for our consideration.

For years the most prominent ones have been ligation, the clamp and cautery, and removal by the ecraseur. This last, as judged by my readings and personal communication with surgeons, has rapidly lost its one-time popularity. This neglect, I think, was a merited one. In the best of hands, the removal of the tumor in this way was occasionally followed by serious, even fatal, hemorrhage. Nor are the vessels involved in the making of the tumor permanently altered in character, in my opinion, as by other methods.

The ligature, undoubtedly, may be regarded as a reliable stand-by. The certainty with which we can assure a sufferer that this operation will restore him to his pristine condition of rectal and anal smoothness, in form and function, is refreshing, as we recall the many instances of surgical disease in which the comfort of such assurance is denied us. Nor do we fail to emphasize, in this connection, its immunity from danger. Certainty of cure and immunity from danger! Surely this combination is sufficient to justify the popularity of this method even in this day of restless search for novelty.

What, then, is the drawback, the objection, to ligation? Why desire other means of cure? Pain and loss of time must result, to a greater or less degree, from the tying of the tumors. I say to a greater or less degree; for carefulness in execution may greatly lessen both of these objectionable features.

If the ligature is thrown around a needlessly large mass of tissue, a needless length of time is required for the separation of the condemned portion, and there must be the corresponding increase in duration of pain. If the ligature is drawn but too loosely, then will there supervene undue inflammation, with the torture of a slow death.

If we leave sensitive skin or membrane to be cut through by ligature, rather than carefully, but effectively, divide them with our knife, the patient must pay the penalty of our sin of omission. More and more care do I find myself bestowing, at each operation, upon the details just set forth, for I find such care secures a liberal reward in lessened suffering and quickened recovery. I endeavor, before operating, to acquaint the patient with what may be expected to follow. Yet I generally find, since I have practiced this increased nicety of detail, that the patient afterwards expresses gratified surprise at his easy escape.

The clamp and cautery I regard as the equal altogether of the foregoing method. It has not come into such general use, however. Not only does it require more apparatus, but the patient cannot tolerate the idea of applying the blazing iron to his precious person in a spot of such tender memory. But aside from these objections, this operation is perhaps the most satisfactory of any when well executed. The Paquelin cautery, if in general use, would render this a more popular method of treatment. This instrument is less formidable in appearance than the blow-furnaces heretofore employed for heating irons, and it is far more efficacious in application. The better plan is to use the clamp and then the cautery after excision; though of course the tumors may be readily destroyed by directly thrusting them through with a cautery point.

The treatment by injection with various substances has received much attention during the past few years. This really suggested this little communication. The injection cure has for some years been blended with quacks and quackish methods. Any potent remedy applied blindly and indiscriminately, without regard to pathology, or to the pathological results that may be the outcome of such application, must show occasional ill result following such application. Such has often been the result in this case.

There is involved in this injection cure, however, a principle that by proper guidance can accomplish much in the line of which we now treat, and that, too, safely and effectually. Instead of searching for, or depending upon, a specific or a magic remedy, we must recognize the principle of cure and know how to secure that principle, or that effect, with certainty, and find where it may or may not be safely and effectively applied.

The object aimed at is the obliteration of the constituent parts of this tumor; that is, of such elements as permit the formation and growth of the hemorrhoid. If the tumors are almost entirely composed of blood-vessels, they are certainly very nearly what is known as erectile growth. But for this enlargement of the vessels, whether arterial or venous, there would be no hemorrhoid. What cell-proliferation there may be depends upon the vascular growth. With this it springs into being, and this destroyed, so, too, dies the tissue that it brought forth.

Then we should convert this erectile growth, first, into what is now known as a sclerosis (or a very similar condition). This done, our object is acccomplished—or as much of it as we may directly do. Nature's processes, to use a common term, may go further and correct this induced pathological condition ; the sclerotic tissue may be absorbed, as the word goes. At any rate, there is no longer space for the vessels to swell and strut this loose, formerly loose structure, and so carry in its train all those processes which, when properly compounded, make up the full-blown hemorrhoid.

In our injections, we should provoke the smallest inflammation that can secure the requisite thickening. If we go too far, the tissues may be entirely destroyed, either at once by the too powerful remedy, or some hours later by the extent and intensity of the inflammation.

If this immediate destruction of hemorrhoidal tissue could be controlled, or would arrest itself, just here we might have cause for congratulation at our precipitate treatment. We find, on the contrary, that the inflammation will extend. We are already aware of the readiness with which the loose tissue around the lower rectum takes on suppurative inflammation; hence, we are not surprised at the result. But if one who is not thus familiar with this tendency be the operator, he will be surprised at having cured the pile by swapping it for a formidable abscess. Such abscess is, of course, apt to terminate in a fistula. This sinus may be comparatively superficial; that is, may either not terminate in the rectum at all, or else very near the anus. This must depend on the location of the tumor injected, or on the extent of the suppuration. Such, then, are the ends we must hold in view: the first, that we may secure the requisite inflammation; second, that we may avoid that violent action which will lead to the evils last set forth. There are many substances which, when injected into the tissue under discussion, will provoke proper inflammation. Indeed, if we aimed at this process alone, that is, without regard to degree, then almost any liquid would answer. It will be observed that I insist upon the virtue of this proper degree of the inflammatory process rather than upon any inherent property of any special agent. Of course, it is to the interest of certain parties to assign the magic virtue to the one particular agent they may use. In such cases, the endeavor is made to take advantage of some astringent or coagulating power possessed by the remedy. Nor is it my intention to deny altogether the special adaptation of certain agents, but rather to hold up the correct principle which, after all, must underlie the action of remedies, even when they may be empirically applied.

There are instances where the action following the injection has been so mild as to scarcely bear the name of inflammation. Most of the symptoms by which we ordinarily recognize that condition may be absent, or so mildly present as to scarcely attract attention. Nevertheless, the "changes of nutrition" must go on, else the results would not be achieved.

The mere constringing of vessels, or of other tissue, will not answer. The mere coagulation of blood would as certainly fall short of the purpose of the injection. This last would of itself lead on to inflammation.

Now to the practical question—the cure of the patient of his hemorrhoids; for he only asks that this be done easily, quickly, safely, permanently. He is anxious to avoid loss of time; he is anxious to avoid an operation. That word is the bug-bear that sends him fleeing from the surgeon too often to some charlatan. If we can fulfill the requirements just set forth, we undoubtedly should do so. There is no question but even the largest of these tumors sometimes yield kindly to injection; their size and age I consider no contra-indication to such treatment.

It is essential that our patient can present himself sufficiently often for us to make the injections at proper intervals. It cannot be done "all at once." If this must be required, then we must adopt one of the other methods which I have recommended.

Further, I have never felt able to so definitely promise a cure as by the other methods, yet, I am not sure but it can be as *certainly* obtained if the patient perseveres as directed; but not always so safely.

The greatest drawback is the fear that some undue inflammation may complicate the treatment. This accident may be avoided by care—not certainly, but almost certainly avoided, I will add.

Above all, I would impress the necessity of avoiding injections of too great strength or two bulky in quantity. These qualities are unnecessary, and would possibly cause either sloughing or that dangerous inflammation already alluded to. The quantity deserves as much consideration as does the strength of the injection. This must vary with the size of the tumor. The judgment of the surgeon must determine this in each case. The mildest fluid, even water itself, used to too great a distention, would cause a hemorrhoid to slough out. I very naturally tried the emptying of the hemorrhoid of its blood by the reversed action of the syringe before injecting. Stress has been laid on this. From my experience, I may say that such attempt at aspiration is not apt to prove successful.

If I mistake not, we have been cautioned against injecting more than one tumor at a time. In a patient in good condition, I do not hesitate to inject more than one, using extra care as to proper dilution and bulk.

The injection of a tumor should not be repeated until all effect of the previous one has passed away. Very often the first is sufficient.

As to the agent used, I have become familiar with the use of carbolic acid, and therefore prefer it. I have found a five or ten per cent. solution sufficient in most instances, and from two to four or six minims sufficient in quantity. However, I am governed more by each case than by any special rule.

It is possible, by observing the principles laid down, to entirely cure even severe hemorrhoids with comparatively little pain or detention. But I especially caution against the evils pointed out. A little extra zealousness or misapplied judgment may cause more inconvenience than your patient had ever felt from his ailment, or more than either the ligature or cautery would probably inflict.

I believe there has been a tendency, in certain quarters, to a too zealous practice of this injection treatment. I believe the popularity it once enjoyed will suffer further diminution because of these occasional complications.

When a severe sloughing or an abscess has occurred, and the suppuration has been extensive around the anus or rectum, the patient is apt to decide that he has had enough of this treatment. Nor is such occurrence calculated to make the surgeon enthusiastic in its praise.

I am not prepared to claim that the operator can ever feel absolutely secure against such misfortune, but he may feel comparatively safe, even comfortably so, by the never-failing exercise of careful conservatism. It is better that we do too little rather than too much.

I took occasion, as I first penned these lines, to make kindly comment upon the long list of cases reported two or three years since by Dr. Kelsey, of New York, congratulating him upon having almost uniformly escaped the accidents just discussed, notwithstanding the large number of cases that had come under his care.

In a little book by the same author, "The Treatment of Hemorrhoids," more recently published, and which I have just had opportunity to examine, I find that he has honestly modified his opinion as to the almost universal safety of these injections. In his later series of cases, he very frequently met sloughing of the tumors, subcutaneous suppurations, and occasionally more extensive abscesses. He also states that he had not been able to predict or prevent an abscess in a given case.

As in his first series published he had been more uniformly successful than I had been, so in his last his misfortunes appear to have crowded more rapidly than mine. It is proper for me to observe that he has practiced this method much more extensively than I have done.

I had already expressed the opinion, which is endorsed in the little work just quoted, that the "injection treatment" had commenced to decline in popularity.

While this treatment has undoubtedly been abused, especially by the class by whom it was first popularized, I repeat that it is the duty of the profession to recognize what of good there is in it, and bring it as nearly as possible to that accuracy of application, or certainty of achieving the beneficial results and avoiding the evil effects, which alone is lacking for establishing it as a standard treatment.

I do not hesitate to say that our greatest degree of safety will be found in using, as already set forth, injections of carbolic acid very largely diluted and in small quantity. Instead of fifteen per cent. as the minimum, I prefer to make it the maximum; and the quantity thrown in I should place at from one to four or five minims, thus determined by the size of the tumor. It should be placed accurately in the tumor and nowhere else. I believe water is a better solvent than oil or glycerine. Even should this quantity, in this strength, fail to satisfactorily disperse the tumor, we may well congratulate ourselves that the mistake was on this side rather than on the side of that over-zeal which eventuates either in sloughing or suppuration, or in both together. There is still opportunity of repetition (though generally not needed).

As to the class of hemorrhoids susceptible of this cure, I should exclude those tumors distinctly external, and also a condition commonly classed with hemorrhoids, in which a highly vascular condition, an enlargement of superficial capillaries is present, and gives rise to trouble by its bleeding rather than by growth. This rather infrequent condition is best met by that time-honored remedy, the application of nitric acid. Almost any hemorrhoid that is a distinct growth, a distinct internal growth, I am certain can be successfully met by injection.

But with all this said, the fact still stands forth that the ligature and the cautery offer safety of action and certainty of cure unattainable by any other device. But with these advantages offered and assured, countless men and women drag through a miserable existence, unable to overcome the repugnance and dread associated with these operations.

We may truly say their fears are groundless, but they are nevertheless present and invincible; and I hold that it is just this class of people, and that other class who cannot obtain time or consent to lose the time required by those plans, to whom we are justified in offering the plan of cure by injection.

In this, as in all operations, we must make an honest statement to the patient as to the probable course and result of the method proposed. If the ligature and cautery appear to him so formidable as to beget an invincible repugnance, and the case, in our judgment, be of a character to admit of this so-called milder practice, I hold we are justified, even required, to afford him what advantages it possesses.

This little paper is intended as a jotting down merely of my views, the result of my own thought and experience, and not especially to add to or dissent from more pretentious articles that have been given to the profession by several authors of eminence.

